# The Brain Selectively Tunes to Unfamiliar Voices during Sleep

**DOI:** 10.1523/JNEUROSCI.2524-20.2021

**Published:** 2022-03-02

**Authors:** Mohamed S. Ameen, Dominik P.J. Heib, Christine Blume, Manuel Schabus

**Affiliations:** ^1^Department of Psychology, Laboratory for Sleep, Cognition, and Consciousness Research, University of Salzburg, 5020 Salzburg, Austria; ^2^Centre for Cognitive Neuroscience, University of Salzburg, 5020 Salzburg, Austria; ^3^Centre for Chronobiology, Psychiatric Hospital of the University of Basel, CH-4002 Basel, Switzerland; ^4^Transfaculty Research Platform Molecular and Cognitive Neurosciences, University of Basel, CH-4055 Basel, Switzerland

**Keywords:** auditory stimulation, EEG, information processing, K-complexes, microarousals, sleep

## Abstract

The brain continues to respond selectively to environmental stimuli during sleep. However, the functional role of such responses, and whether they reflect information processing or rather sensory inhibition, is not fully understood. Here, we present 17 human sleepers (14 females) with their own name and two unfamiliar first names, spoken by either a familiar voice (FV) or an unfamiliar voice (UFV), while recording polysomnography during a full night of sleep. We detect K-complexes, sleep spindles, and microarousals, and assess event-related and frequency responses as well as intertrial phase synchronization to the different stimuli presented during nonrapid eye movement (NREM) sleep. We show that UFVs evoke more K-complexes and microarousals than FVs. When both stimuli evoke a K-complex, we observe larger evoked potentials, more precise time-locking of brain responses in the delta band (1–4 Hz), and stronger activity in the high frequency (>16 Hz) range, in response to UFVs relative to FVs. Crucially, these differences in brain responses disappear completely when no K-complexes are evoked by the auditory stimuli. Our findings highlight discrepancies in brain responses to auditory stimuli based on their relevance to the sleeper and propose a key role for K-complexes in the modulation of sensory processing during sleep. We argue that such content-specific, dynamic reactivity to external sensory information enables the brain to enter a sentinel processing mode in which it engages in the important internal processes that are ongoing during sleep while still maintaining the ability to process vital external sensory information.

**SIGNIFICANCE STATEMENT** Previous research has shown that sensory processing continues during sleep. Here, we studied the capacity of the sleeping brain to extract and process relevant sensory information. We presented sleepers with their own names and unfamiliar names spoken by either an FV or a UFV. During NREM sleep, UFVs elicited more K-complexes and microarousals than FVs. By contrasting stimuli that evoked K-complexes, we demonstrate that UFVs evoked larger, more synchronized brain responses as well as stronger power at high frequencies (>16 Hz) relative to FVs. These differences in brain responses disappeared when no K-complexes were evoked. Our results suggest a pivotal role for K-complexes in the selective processing of relevant information during NREM sleep.

## Introduction

During sleep, the brain continues to respond to auditory stimuli in a selective fashion (Portas et al., 2000; Andrillon et al., 2016; Blume et al., 2017, 2018). Previous studies have demonstrated, for instance, that the subject's own name (SON) evokes stronger brain responses than other names during sleep (Oswald et al., 1960; Perrin et al., 1999; Pratt et al., 1999). Blume et al. (2018) showed that during all stages of sleep, brain responses to SON and other unfamiliar names (UNs) did not differ; however, names uttered by an unfamiliar voice (UFV) evoked stronger brain responses compared with a familiar voice (FV).

The discrepancy in brain responses to different stimuli implies the presence of an initial, presumably low-level, sensory processing during sleep that enables the brain to differentiate between sensory signals (Blume et al., 2018). However, knowledge about the functions of such responses is still lacking. That is, the selective brain responses to specific sounds during sleep might reflect inhibitory processes that protect sleep from disruptions. Conversely, they might indicate further, higher level processing that ensures the connectedness of the sleeping brain to the surrounding.

In this study, we investigated the purpose of such selective brain responses to sounds presented during nonrapid eye movement (NREM) sleep. We focused on sleep-specific events that have been previously linked to information processing, sensory inhibition, or both. That is, we focused on three cardinal sleep-specific electroencephalography (EEG) events, namely, the K-complex (KC), sleep spindles, and microarousals.

KCs are ∼1 Hz oscillations and a hallmark of Stage 2 NREM (N2) sleep (Loomis et al., 1938; Colrain, 2005; Halász, 2005). KCs occur either spontaneously or in response to sensory stimuli. Spontaneous KCs appear in the EEG signal as a well-defined sharp negative wave followed by a positive component with a total duration of at least 0.5 s (Rechtschaffen and Kales, 1968; Hori et al., 2001). Following sensory perturbation, KCs appear to have two main components, a sharp negative (N) deflection at ∼550 ms (N550) followed by a longer-lasting positive (P) wave at ∼900 ms (P900; Bastien and Campbell, 1992; Cote et al., 1999; Colrain, 2005; Halász, 2005). Some studies have considered an early positive peak that appears ∼200 ms (P200; Laurino et al., 2014, 2019) and another negative peak ∼350 ms (N350; Bastien and Campbell, 1992; Cote et al., 1999) to be parts of the KC, albeit these components can occur without a KC being elicited. Relevant stimuli have a higher propensity to trigger KCs (Halász, 2005). Theories suggest that KCs can serve both sleep-protecting as well as arousal-inducing processes (Halász, 2005; Jahnke et al., 2012; Forget et al., 2011; Laurino et al., 2014; Blume et al., 2017, 2018; Legendre et al., 2019; Latreille et al., 2020).

Sleep spindles are also characteristic of N2 sleep. Spindles are thalamocortical oscillations of 11–15 Hz that last ∼0.5–2 s (De Gennaro and Ferrara, 2003; Fernandez and Lüthi, 2020) and can be triggered by sensory stimuli (Antony and Paller, 2017). They have been repeatedly shown to inhibit sensory processing during sleep (McCormick and Bal, 1994; Schabus et al., 2012; Blume et al., 2018; Fernandez and Lüthi, 2020). However, some work challenges this notion (Sela et al., 2016) and even associates spindles with the processing of memory-related sounds presented during NREM sleep (Cairney et al., 2018).

Finally, microarousals are abrupt shifts in the EEG signal toward theta, alpha, and/or high beta (>16 Hz) frequencies (Halász et al., 1979; American Sleep Disorders Association, 1992; Halász et al., 2004) that appear in all sleep stages and are considered windows of information processing during sleep (Halász et al., 2004; Halász, 2005; dos Santos Lima et al., 2019). Micro-arousals are usually preceded by KCs (Colrain, 2005; Halász, 2005), yet they have been shown to be correlated with a lower incidence of sleep spindles in the preceding 10 s of EEG signal (Ehrhart et al., 1981).

Here, we reanalyzed the dataset used in Blume et al. (2018), who recorded polysomnography while presenting SONs and two UNs spoken by either an FV or a UFV during a whole night of sleep. We detected KCs, spindles, and microarousals in response to these sounds during NREM sleep and hypothesized that the selective auditory-evoked responses support the extraction and processing of relevant sensory information.

## Materials and Methods

### Participants

We recruited 20 healthy participants with no reported history of neurological or psychological problems as well as no reported sleep disorders. However, one participant dropped out after the adaptation night, and we had to exclude two participants because of technical problems during EEG acquisition. Therefore, we performed the analyses we report here on 17 participants (14 females) with a median age of 22.6 ± 2.3 years. Before beginning the experiment, all participants signed written informed consents. The experiment was approved by the Ethics Committee of the University of Salzburg.

### Experimental design

**Figure 1.**
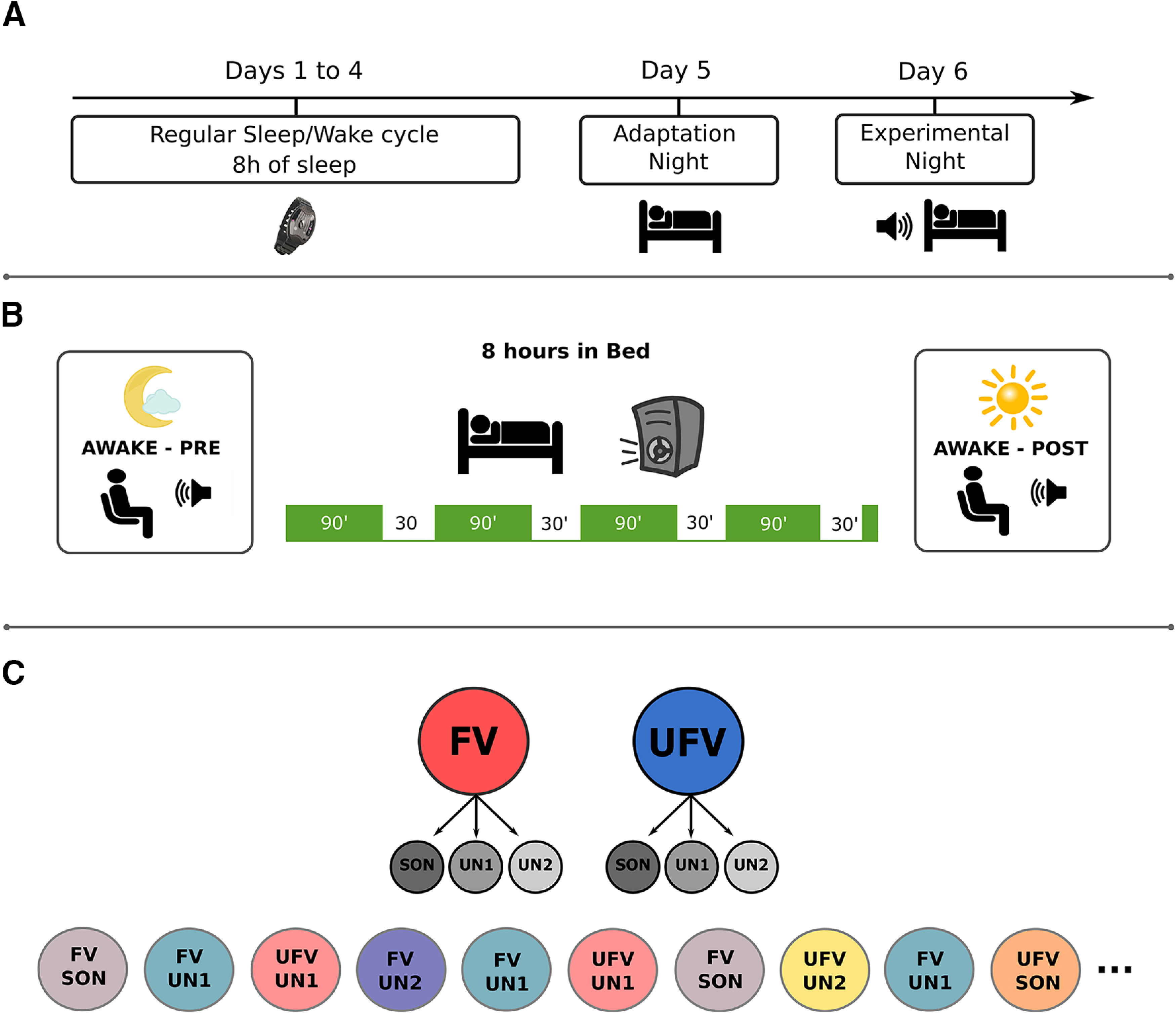
Experimental design. ***A***, Protocol. Participants were invited for an initial screening interview during which they were given the wrist-worn actigraphy and advised to keep a regular sleep-wake cycle. Participants slept in the sleep laboratory during two nights, an adaptation night during which polysomnography (PSG) was recorded but no stimuli were presented, and an experimental night during which we recorded PSG and presented auditory stimuli throughout the night. ***B***, Procedure of the experimental night. Participants slept for ∼8 h with PSG and auditory stimulation. All participants went through one stimulation session in the evening before sleep (Awake - pre) and one in the morning after waking up (Awake- post); however, these sessions are irrelevant to the current analysis. The auditory stimuli started directly after participants went to bed and continued for 90 min, then paused for ∼30 min to allow for a period of undisturbed sleep. This cycle was repeated four times for the whole duration of the night. ***C***, Top, Stimuli. We presented the subject's own name (SON) and two unfamiliar names (UN1 and UN2) spoken by either a familiar voice (FV) or an unfamiliar voice (UFV). Bottom, An exemplary sequence of stimulus presentation. Stimuli were presented in a pseudorandom order; each stimulus was presented 690 times, and the interstimulus intervals ranged between 2800 and 7800 ms.

Before the start of the experiment, participants were advised to maintain a regular sleep/wake cycle (∼8 h of sleep) for at least 4 d, which we monitored via actigraphy (Fig. 1*A*). Subsequently, participants spent two nights in the sleep laboratory of the University of Salzburg. The first night was an adaptation night, during which we recorded polysomnography (PSG) data with no auditory stimulation. The second night was an experimental night, during which we recorded PSG data while presenting sounds via loudspeakers throughout the night. In both nights, participants were tested during wakefulness before and after sleep. Briefly, the wakefulness testing consisted of two sessions, a passive-listening session and an active-listening session. Passive listening entails the participants' listening to the repeatedly presented auditory stimuli, whereas active listening means they had to count the number of presentations of one specific stimulus chosen by the experimenters. Before the wakefulness testing, participants were stimulated with either a bright (blue enriched) light or an inactive (sham) light for 1 h. The order of the light-stimulation conditions was counterbalanced between the adaptation and the experimental nights across participants. However, the light condition is irrelevant to this study as we grouped our data over both light conditions. For the purpose of this article, we focus primarily on the sleep part of the experimental night. For more details on the wakefulness part of the experiment, please refer to Blume et al. (2018). During the experimental night, participants went to bed around their habitual bedtime (8:30 P.M. to 11:30 P.M.). Time in bed (TIB) was ∼8 h. After 8 h TiB, we waited for light NREM or REM sleep before waking up the participants (median sleep duration = 480 ± 2.5 min). The auditory stimulation started directly after participants went to bed and continued throughout the whole night. We presented auditory stimuli continuously for 90 min (Stimulation periods) then paused the presentation for 30 min (No-stimulation periods) to allow for periods of undisturbed sleep. This resulted in a 120 min cycle that we repeated four times throughout the night (Fig. 1*B*).

### Stimuli

We presented six different auditory stimuli (Fig. 1*C*) that we personalized for each participant. The stimuli were the SON and two UNs spoken by either an FV or a UFV. An FV was the voice of someone close to the participant, for example, one of the parents. An UFV was the voice of someone unknown to the participant. We did not control for the sex of the voices, but they were matched; that is, the familiar and unfamiliar voices were always both either male or female. We chose UNs that matched the SON in the number of syllables and the frequency of occurrence in the population. The volume for stimulus presentation was adjusted individually for each participant so that the participant could clearly hear the stimulus and still be able to fall asleep. Each stimulus was presented 690 times, and the mean duration was 808 ± 110 ms. Stimuli were presented in a pseudorandom order, and no stimulus was presented twice in a row. The interstimulus intervals during sleep were jittered between 2800 and 7800 ms in 500 ms steps. Stimulus preprocessing, that is, denoising and normalization, was performed using Audacity software (https://audacityteam.org/). Stimulus delivery was controlled by MATLAB (MathWorks).

### Brain data acquisition

We recorded ongoing brain activity using a high-density EEG 256-channel GSN HydroCel Geodesic Sensor Net (Electrical Geodesics) and a Net Amps 400 amplifier. The PSG recordings included two electrooculography (EOG) and two chin electromyography (EMG) channels. Data were acquired at a sampling rate of 250 Hz, and Cz served as the online reference.

### Sleep staging

Sleep staging was performed on 30 s epochs using the computer-assisted sleep classification system developed by the SIESTA Group (Somnolyzer 24 × 7; Anderer et al., 2005, 2010) following the standard criteria recommended by the American Association for Sleep Medicine (Iber, 2007). We have previously shown that the level of agreement between this algorithm and expert human scorers is similar to the level of agreement between human experts (Ameen et al., 2019).

### The detection of sleep microstructures

#### KC detection

**Figure 2.**
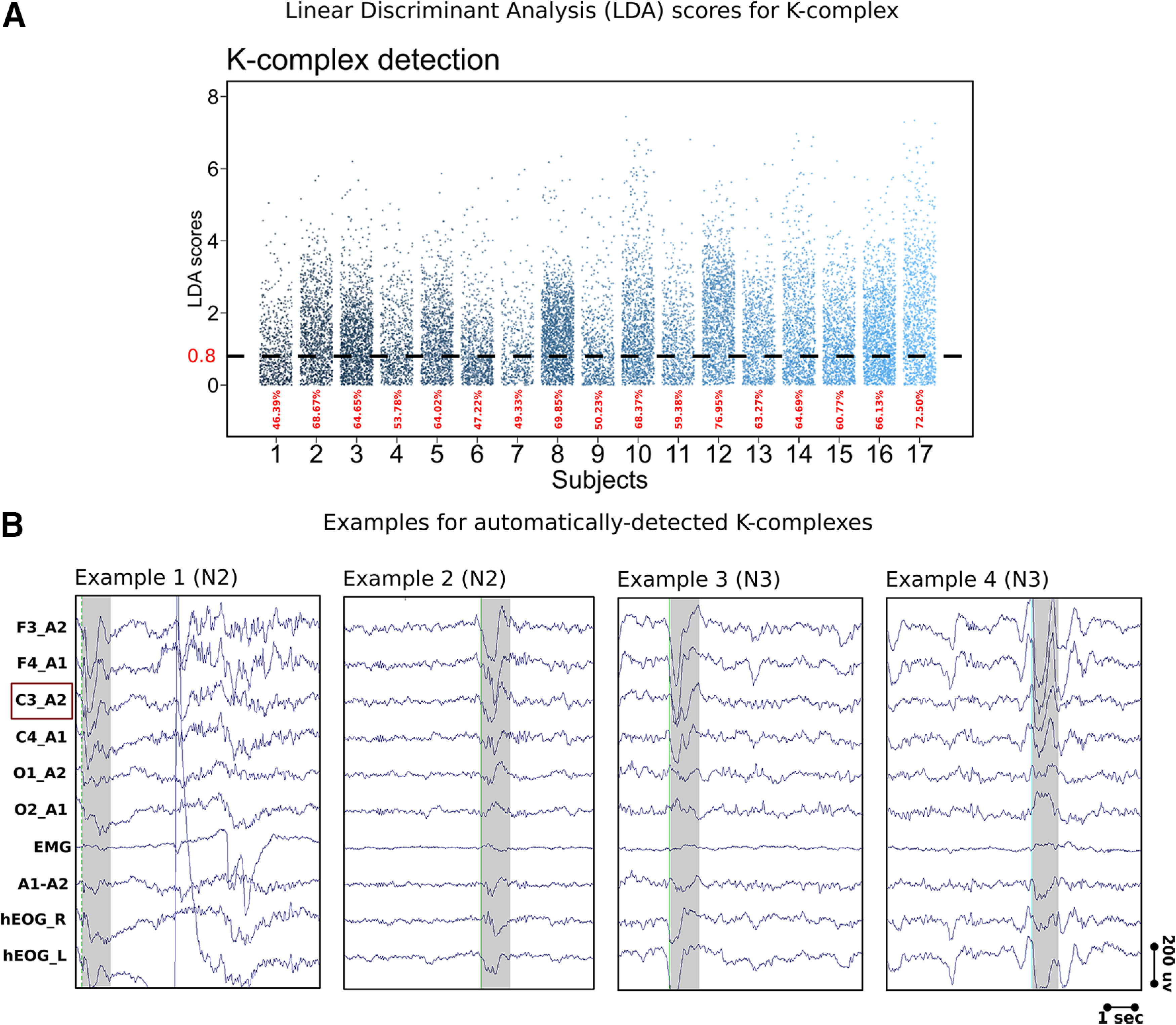
K-complex detection and examples. ***A***, LDA scores for all detected K-complexes for all subjects. The dashed line represents our minimum cutoff at an LDA value of 0.8, which is the threshold used for the selection of reliable K-complex events. Bottom, The percentage of the events used in our analyses from all the detected events for each subject are indicated in red. ***B***, Examples of the detected K-complexes at channel C3 referenced to the contralateral mastoid. We show the standard EEG montage that we used for sleep staging as well as event detections. Specifically, we used the following channels (from top to bottom): F3, F4, C3, C4, O1, and O_2_ referenced to the contralateral mastoid. Moreover, we show one EMG and two EOG channels (hEOG_R and hEOG_L) channels as well as the average of both mastoids (A1-A2). Examples 1 and 2 show K-complexes detected in N2 sleep from one subject, whereas examples 3 and 4 are K-complexes detected during N3 in a different subject.

We detected KCs automatically with a wavelet-detection algorithm that was developed by the SIESTA Group. The development and validation procedures have been described in detail in Parapatics et al. (2015) and Schwarz et al. (2017), respectively. Briefly, 12 experienced human scorers visually scored KCs in 873 epochs of 10 min of PSG recordings from 189 control subjects and 90 patients. The features of the visually scored KCs were used to set the criteria for detection as well as to create a template KC that worked as a gold standard for the automatic detection. The detection itself is a two-step process; first, the algorithm detects possible KCs via an approach that combines a matched-filtering detection method and a slow-wave detection method (Woertz et al., 2004). Accordingly, the detection criteria for possible KCs were the following: (1) a minimum negative-to-positive peak-to-peak amplitude of 50 µV and (2) a duration between 480 and 1500 ms. Second, all possible KCs are matched to the prototypical KC template via wavelet analysis, and the results are submitted to a linear discriminant analysis (LDA) to select only real KCs. For our analysis, we considered real KCs to be events that have an LDA score (how likely a specific EEG segment is a KC) of 0.8 or higher. This LDA score corresponds to 61.87% ± 9.14 of all detected KCs and a mean correlation to the template of 0.87 ± 0.007 over all subjects and is a compromise between reliable detections and a sufficient number of detected events for our analyses. Note that an LDA score of 1.7 reflects 98% detection specificity. Before running the detection algorithm, raw data were downsampled to 128 Hz and rereferenced to the contralateral mastoid. We detected KCs at C3 and C4. We only report results from C3 as the detections were similar between C3 and C4. We only considered events that occurred during N2 and N3 (Slow-wave) sleep and fulfilled the standard criteria for KC detection (Rechtschaffen and Kales, 1968; Hori et al., 2001). For N3 detections, however, we applied a stricter amplitude criterion as we only selected events with a peak-to-peak amplitude of 75µV or higher (Cote et al., 1999; Nir et al., 2011). We marked the start of KC events as the point of the negative-going zero crossing of the signal before the negative peak. We defined evoked KCs as those events that occurred (started) in the 2000 ms poststimulus-onset window. Figure 2 demonstrates the LDA distribution of the detected KCs and contains some examples of the detected events.

#### Spindle detection

Sleep spindles were detected using an algorithm developed by the SIESTA Group (AskAnalyzer; Gruber et al., 2015). First, we filtered the raw data between 11 and 16 Hz and then detected spindle events at frontal (F3, F4, Fz) and central (C3, C4, Cz) channels rereferenced to the average of mastoids. We used the criteria described in Schimicek et al. (1994).Only events with an amplitude >12 µV and a duration between 500 and 2000 ms were considered. Further validation of the detected spindles was done using LDA in which the detected spindles were compared with a template that is generated based on the visual scoring of sleep spindles in 8730 min of PSG data from 189 healthy participants and 90 participants with sleep disorders. For our analyses, we only considered events that occurred during N2 and N3 sleep with an LDA score of 1.7 or higher (Anderer et al., 2005). To identify the frequency of each spindle event, the algorithm preforms period-amplitude analysis of the bandpass-filtered signal in the time domain. We subdivided spindles into slow (11–13 Hz) and fast (13–15 Hz) spindles based on the dichotomy in their topography and functions (Schabus et al., 2007). We report results from fast spindles detected at C3 and slow spindles detected at F3.

#### Microarousal detection

**Figure 3.**
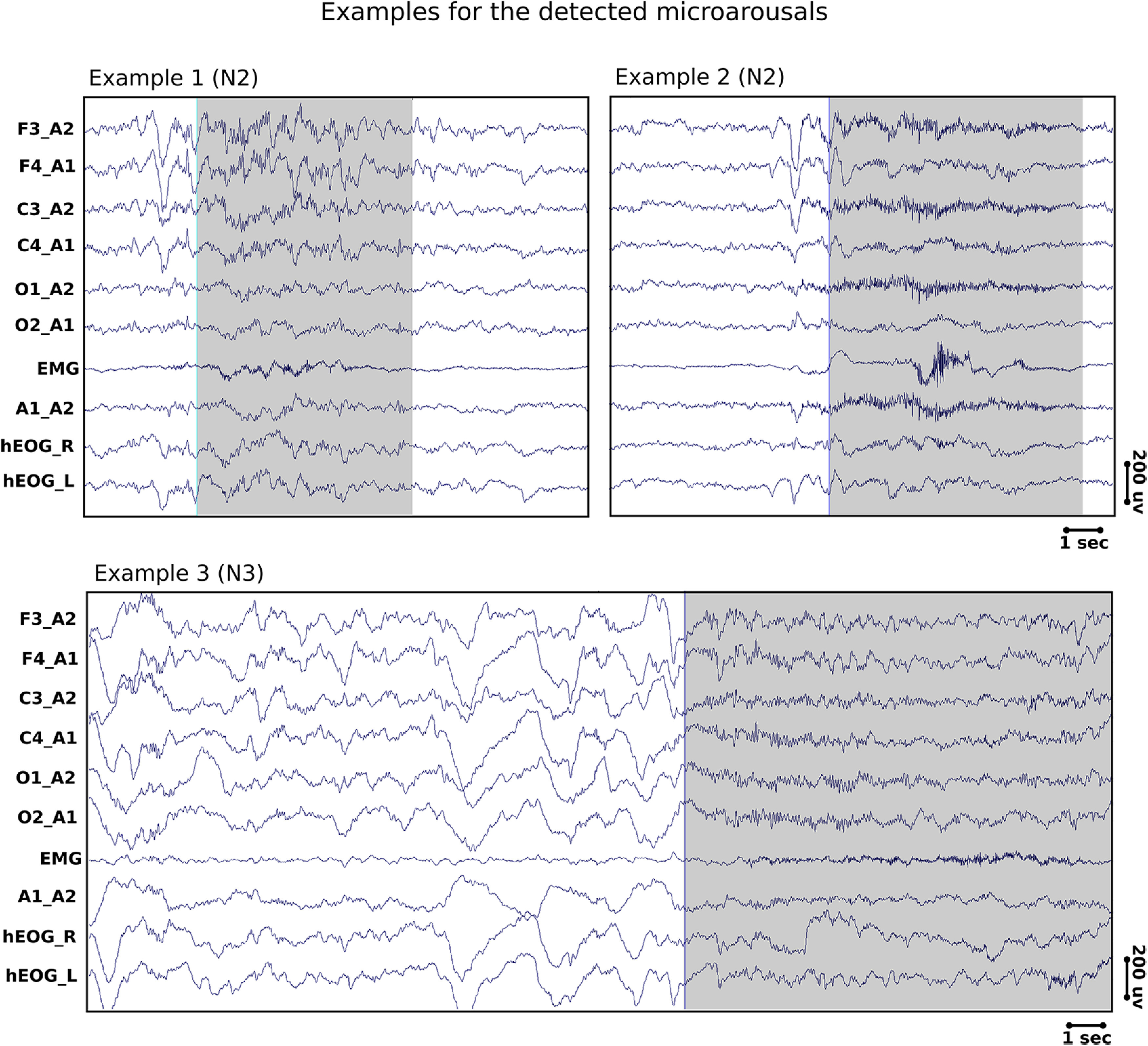
Examples of the detected microarousals. We show the standard EEG montage that is used for sleep staging as well as event detections. Specifically, we used the following channels (from top to bottom): F3, F4, C3, C4, O1, and O_2_ referenced to the contralateral mastoid. Moreover, we show one EMG and two EOG channels (hEOG_R and hEOG_L) as well as the average of both mastoids (A1-A2). Examples 1 and 2 are events detected in N2 sleep from one subject. Example 2 shows a microarousal that is preceded by a K-complex. Example 3 is from an N3 epoch from a different subject.

We detected microarousals semiautomatically using an algorithm developed by the SIESTA Group, which has been described in detail in Anderer et al. (2010). Briefly, the algorithm was developed using the scoring of 12 PSG recordings by six independent experts. It incorporates information from central and occipital channels. First, the algorithm compares the absolute and relative power of nine frequency bands including theta, alpha, and high beta (>16 Hz) frequencies between a 3 s test window and a moving 10 s baseline via a series of LDA separately for each channel. Second, the start and end of each event are determined by combining the posterior probabilities of all channels so that the number of microarousals per total sleep time is the same for both automatic and visual detections. Although EMG increases are not necessary for the identification of microarousals that occur during NREM sleep, some of the detected microarousals showed a concurrent increase in the amplitude of the EMG signal; however, this increase in EMG activity was not time locked to the high-frequency shifts of the EEG signal. The algorithm detects microarousals in all sleep stages; however, for the purpose of this study we selected microarousals that occurred during N2 and N3 only (Fig. 3, detected microarousals).

#### The detection of transient microstates (micromeasures of alertness)

The detection of sleep microstates provides a more fine-grained scoring that detects transient changes in sleep architecture in 4 s epochs rather than 30 s, using an algorithm described in Jagannathan et al. (2018). The algorithm uses the Hori scale (Tanaka et al., 1996) to classify the epochs into either awake, drowsy (N1), or N2 based on the signal from a subset of 14 electrodes distributed over frontal, central, parietal, occipital, and temporal regions. We extracted stimuli from N2 sleep only and removed stimuli with interstimulus intervals of <4000 ms. Then we filtered the data between 0.1 and 30 Hz before running the algorithm on an equal number of epochs in all conditions.

### EEG preprocessing and analyses

#### Preprocessing

We performed all the preprocessing steps in EEGLAB, version 14.1.1b (Delorme and Makeig, 2004). First, we excluded face and neck channels and downsampled the raw data from 183 EEG channels to 128 Hz. Then, we filtered the data between 0.1 and 40 Hz using a Butterworth bandpass filter. We performed bad channels rejection and interpolation as well as rereferencing to an average reference using the PREP pipeline described in Bigdely-Shamlo et al. (2015). Finally, we performed independent component analysis using the adaptive mixture independent component analysis toolbox and visually detected and discarded eye and muscle artefacts.

#### Event-related analysis

We epoched the preprocessed data into 3000 ms trials (−1000 to 2000 ms relative to stimulus onset). For each participant, we converted ERPs into percent power change relative to the 500 ms prestimulus-onset window using the formula (Data – mean baseline values)/mean baseline values.

#### Time-frequency analysis

Time-frequency representations (TFRs) were computed over 8000 ms epochs, (−4000 to 4000 ms relative to stimulus onset). We choose relatively long epochs to avoid edge artifacts because of the transformation. We calculated TFRs by applying a 500 ms hanning window as taper on frequencies from 0.5 to 30 Hz in 0.5 Hz frequency steps and 5 ms temporal steps. Similar to ERPs, we converted participant-specific TFRs into percent power change relative to the 500 ms prestimulus-onset window.

#### Intertrial phase coherence estimate

Following time-frequency transformation, we extracted the complex Fourier coefficient for each channel, frequency, and time point in every single trial. Then we computed the phase angles in each trial before finally averaging the single-trial intertrial phase coherence (ITPC) values over all trials per subject. We performed all analyses in FieldTrip (Oostenveld et al., 2011; https://fieldtriptoolbox.org).

### Statistical analyses

For all of our analyses, we randomly selected an equal number of events/epochs per condition from N2 and N3. Evoked events were defined as events that are detected by our algorithms in the 2000 ms poststimulus-onset window. Because of violations to the assumptions of parametric testing, we applied rank-based nonparametric tests using the nparLD function implemented in the nparLD package available in R (Noguchi et al., 2012). We report ANOVA-type statistics (ATS), *p* values (α = 0.05, two sided), as well as effect sizes using relative treatment effects (RTE). Generally, RTEs represent the probability of the values from the whole dataset being smaller than a randomly chosen observation from the respective group. Therefore, RTE values range between zero and one. An RTE value of 0.5 means no effect. The higher the RTE value of one condition, the higher the probability that a randomly chosen value from that condition is larger than that randomly drawn from the whole dataset, and vice versa. When applicable, we performed *post hoc* tests via the nparLD function with Bonferroni's correction for multiple comparisons. For repeated measures at different time points, we performed nonlinear mixed regression via generalized linear mixed models (GLMMs) implemented in the glmer function of the lme4 package in R (Bates et al., 2015). Both KC and microarousals were non-normally distributed. For K-complexes, we used a GLMM with a Poisson distribution. For microarousals, because of the presence of a notable amount of zero counts, we used the zero-inflated Poisson distribution implemented in the the pscl package in R (Zeileis et al., 2008). We added our subjects as effects with random intercepts and slopes. We report the estimates of the fixed effects (B̂) and their standard errors, *z* values, and *p* values. We performed *post hoc* interaction tests using marginal means estimates as implemented in the emmeans package in R with Tukey's correction for multiple comparisons, and we report Cohen's *d* effect sizes.

For the more temporally resolved analysis to compare between the latencies of the detected events, we binned the 2000 ms poststimulus intervals into bins of 100 ms, then we calculated the mean of the number of events in each bin for each subject and condition, before finally submitting these results to the permutation analysis in FieldTrip. The choice of the bin size is a compromise between a meaningful temporal resolution and a sufficient statistical power.

For ERP, TFR, and ITPC analyses, we selected equal numbers of epochs per condition (66.47 ± 56.25) for each subject. For this analysis, we averaged the signal from six frontal channels (F3, F4, F7, F8, Fcz, and Fz), using a time window from −500 to 2000 ms relative to stimulus onset. We calculated the grand average over all subjects in each condition before submitting the results to the nonparametric cluster-based permutation analysis in FieldTrip (Maris and Oostenveld, 2007). We performed two-sided paired-sample *t* tests followed by Monte Carlo's approximation with 5000 permutations (cluster-alpha = 0.05 and critical alpha = 0.025). We report the sum of the *t* values (∑*t*), as well as Cohen's *d* effect sizes calculated over all possible permutations, channels, time points, and frequencies in the cluster.

## Results

### Auditory stimulation influenced sleep microstructure but not macrostructure

**Figure 4.**
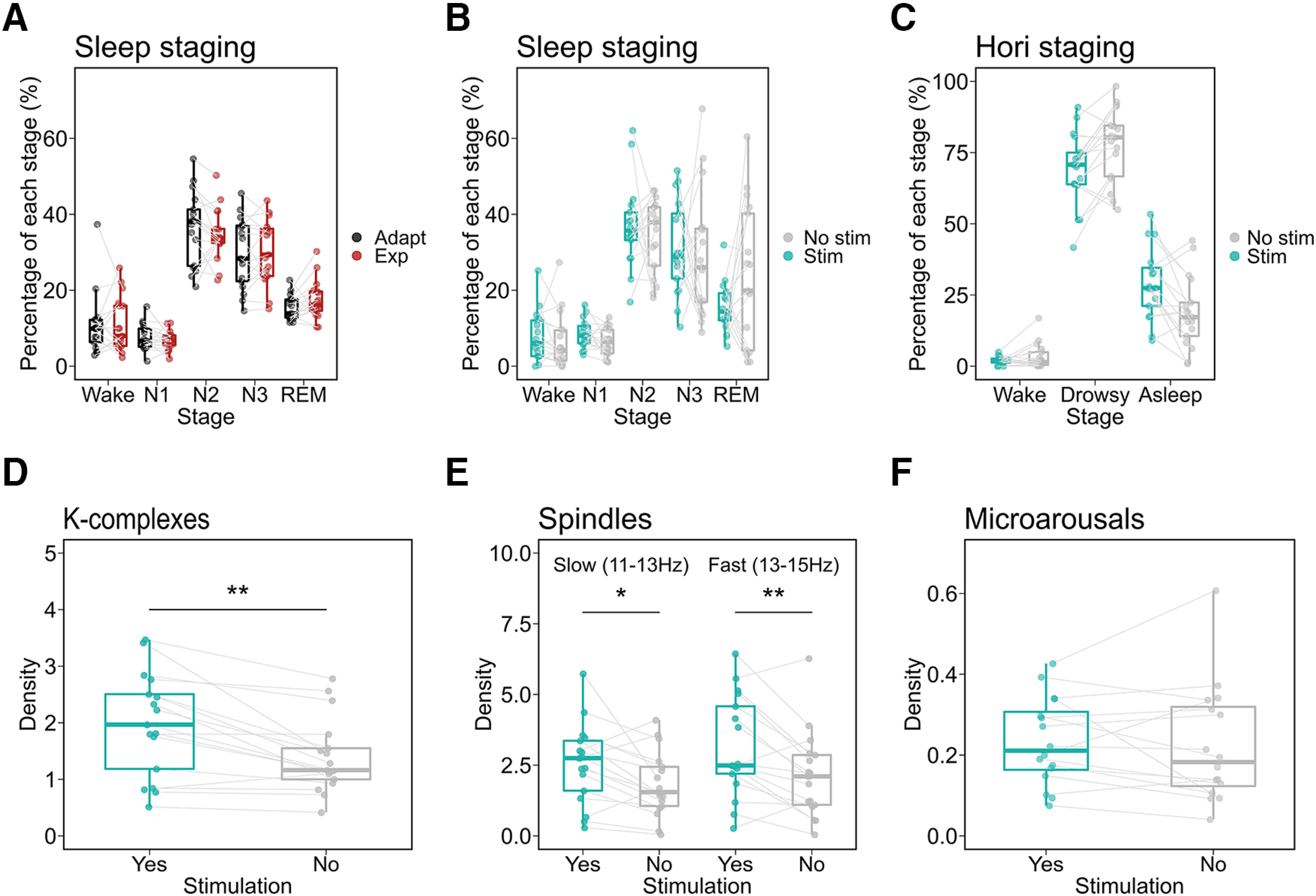
Auditory stimulation influenced sleep microstructure but not macrostructure. In each participant, we selected equal numbers of epochs for all conditions. ***A***, Difference in sleep architecture, that is, the distribution of sleep stages, between the adaptation and the experimental night. To account for the effect of sleep-onset latency, we discarded all epochs that preceded the first N1 epoch. We found no difference between the architectures of both nights. ***B***, Difference in sleep architecture between stimulation and no-stimulation periods during the experimental night. The experimental night consisted of periods of continuous auditory stimulation and periods of no stimulation. We found no difference in the sleep architecture between those periods. ***C***, Microstate differences between the stimulation and the no-stimulation periods. To circumvent the poor temporal resolution of the classical 30-second sleep stages, we opted for a more time-resolved analysis of sleep stages based on the Hori scoring system. We found a higher number of sleep epochs and lower number of drowsy epochs during the stimulation periods, suggesting deeper sleep during auditory stimulation. ***D***–***F***, A comparison of the densities of sleep microstructure between stimulation and no-stimulation periods. K-complex density during the stimulation periods is higher than that in the no-stimulation periods (***D***). Slow and Fast spindles densities are higher during the stimulation periods (***E***). Microarousal density, however, did not differ between stimulation and no-stimulation periods (***F***). Box plots show the median and the whiskers depict the 25% and the 75% quartiles. Each dot/triangle represents one participant in one condition. *p < 0.05, **p < 0.001.

First, we assessed the effects of auditory stimulation on sleep macrostructure. We found that auditory stimulation during sleep does not influence sleep macrostructure. That is, we found no change in sleep macrostructure from the adaptation to the experimental night (Fig. 4*A*). Specifically, we found an effect of *Stage* (*ATS(2.61)* = 62.75, *p* < 0.001, *RTE_wake_* = 0.24, *RTE_N1_* = 0.26, *RTE_N2_* = 0.79, *RTE_N3_* = 0.70, *RTE_REM_* = 0.51), no effect of the *Night* (*ATS(1)* = 0.04, *p* = 0.84, *RTE_Nostim_* = 0.48, *RTE_stim_* = 0.52), and no interaction *Night* × *Stage* (*ATS(2.56)* = 0.75, *p* = 0.51). Similarly, during the experimental night, we found no difference in sleep macrostructure between periods of stimulation and periods of no stimulation (Fig. 4*B*). We found a main effect for *Stage* (*ATS(2.51)* = 53.30, *p* < 0.001, *RTE_wake_* = 0.28, *RTE_N1_* = 0.18, *RTE_N2_* = 0.82, *RTE_N3_* = 0.74, *RTE_REM_* = 0.47). There was no effect of the *Stimulation* (*ATS(1)* = 3.36, *p* = 0.07, *RTE_ADAPT_* = 0.5, *RTE_EXP_* = 0.5), and no interaction *Stimulation* × *Stage* (*ATS(2.08)* = 0.68, *p* = 0.52). We then opted for a more time-resolved analysis of sleep stages based on the Hori scoring system (Tanaka et al., 1996; Jagannathan et al., 2018), which uses 4 s epochs instead of the classical 30 s staging. Again, we found a main effect for *Stage* (*ATS(1.64)* = 146.26, *p* < 0.001, *RTE_wake_* = 0.28, *RTE_N1_* = 0.18, *RTE_N2_* = 0.82, *RTE_N3_* = 0.74, *RTE_REM_* = 0.47). However, there was no effect of the *Stimulation* (*ATS(1.69)* = 3.36, *p* = 0.19, *RTE_ADAPT_* = 0.5, *RTE_EXP_* = 0.5), but a significant interaction *Stimulation* × *Stage* (*ATS(1.47)* = 8.17, *p* = 0.02). *Post hoc* pairwise test with Bonferroni's correction for multiple comparisons revealed that auditory stimulation resulted in a higher number of sleep epochs (*ATS(1)* = 12.84, *p* < *0.001*, *RTE_stim_* = 0.62, *RTE_nostim_* = 0.38) and a lower number of drowsy epochs (*ATS(1)* = *10.88*, *p* < *0.001*, *RTE_stim_* = 0.4, *RTE_nostim_* = 0.6), suggesting even deeper sleep during stimulation periods.

On the level of sleep microstructure, we compared the densities, that is, the numbers of events per minute of N2 and N3, of KCs, slow and fast spindles, as well as microarousals between stimulation and no-stimulation periods. We show that auditory stimulation significantly increased the densities of KCs (Fig. 4*D*; *ATS(1)* = 19.68, *p* < 0.001, *RTE_stim_* = 0.59, *RTE_nostim_* = 0.41) and spindles (Fig. 4*E*; slow: *ATS(1)* = 19.68, *p* < 0.001, *RTE_stim_* = 0.58, *RTE_nostim_* = 0.42 – *fast: ATS(1)* = 8, *p* = 0.005, *RTE_stim_* = 0.58, *RTE_nostim_* = 0.42). However, the increase in the density of microarousals did not reach statistical significance (Fig. 4*F*; *ATS(1)* = 1.01, *p* = 0.31, *RTE_stim_* = 0.53, *RTE_nostim_* = 0.47).

**Figure 5.**
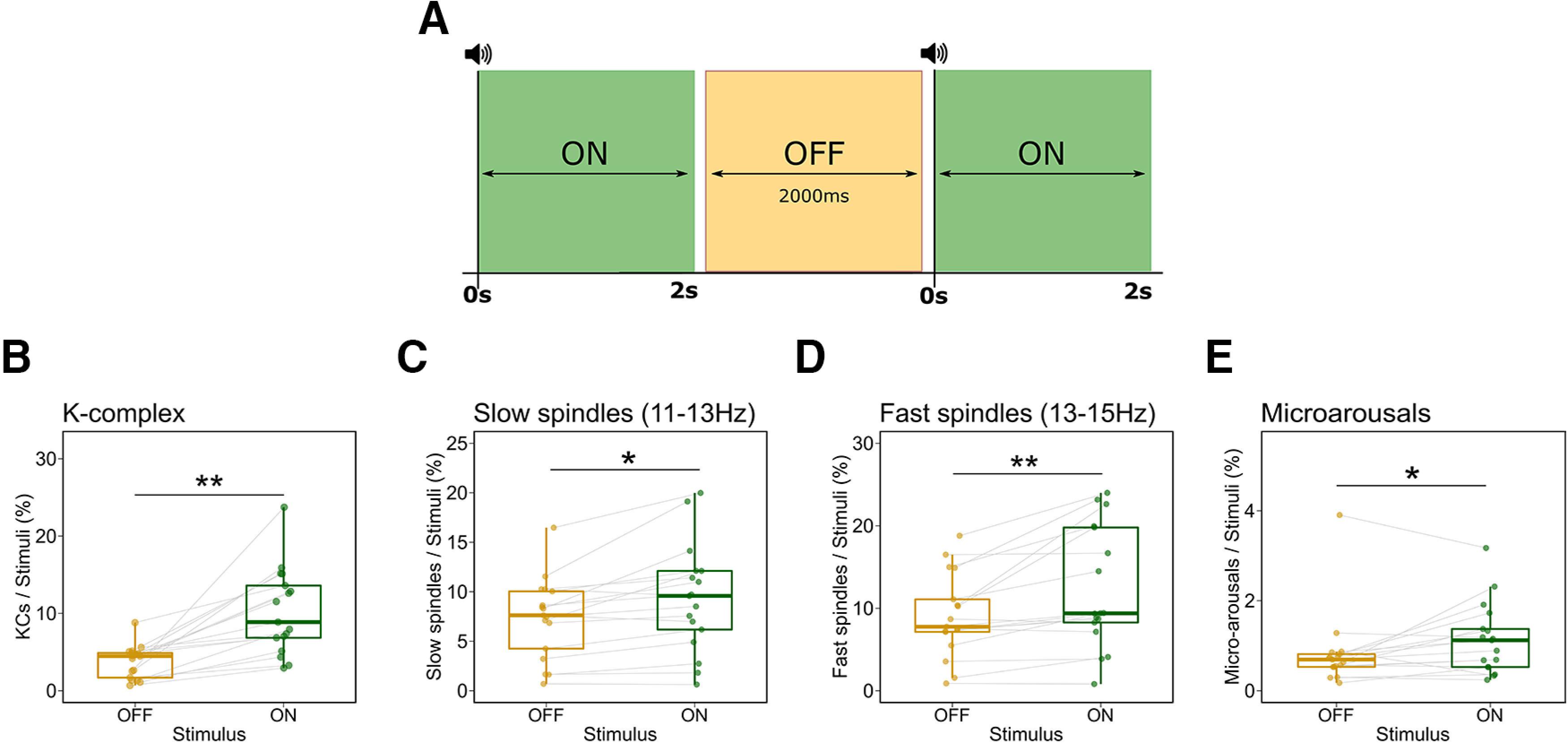
Auditory stimulation increased the occurrence sleep microstructures. ***A***, A graphical illustration of the stimulus-ON and stimulus-OFF windows used for the analysis. We detected events in the 2000 ms poststimulus window (Stimulus-ON, green), as well as during 2000 ms prestimulus windows that are at least 2000 ms after the last stimulus (Stimulus-OFF, orange). ***B–E***, The numbers of (***B***) K-complexes, (***C***) slow spindles, (***D***) fast spindles, as well as (***E***) microarousals were significantly higher during the stimulus-ON than the stimulus-OFF periods. The *y*-axis depicts the percentage of epochs in which events were detected. Box plots show the median, and the whiskers depict the 25% and the 75% quartiles. Each dot/triangle represents one participant in one condition. *p < 0.05, **p < 0.001.

Next, we performed a more temporally resolved analysis of the auditory-induced changes in sleep microstructure. Specifically, we compared the numbers of KCs, spindles, and microarousals between Stimulus-ON and Stimulus-OFF periods during the experimental night. Stimulus-ON periods are 2000 ms poststimulus intervals (0–2000 ms relative to stimulus onset), whereas stimulus-OFF periods are 2000 ms intervals that start at least 2000 ms after the onset of the previous stimulus and during which no sounds are presented (Fig. 5*A*). We found significantly higher numbers of KCs (*ATS(1)* = 54.77, *p* < 0.001, *RTE_ON_* = 0.68, *RTE_OFF_* = 0.32), slow spindles (*ATS(1)* = 9.13, *p* = 0.002, *RTE_ON_* = 0.57, *RTE_OFF_* = 0.43), fast spindles (*ATS(1)* = 20.16, *p* < 0.001, *RTE_ON_* = 0.55, *RTE_OFF_* = 0.45), and microarousals (*ATS(1)* = 8.45, *p* = 0.003, *RTE_ON_* = 0.58, *RTE_OFF_* = 0.42) in the stimulus-ON than in the stimulus-OFF periods (Fig. 5*B*–*E*).

### The brain responds selectively to unfamiliar voices during NREM sleep

**Figure 6.**
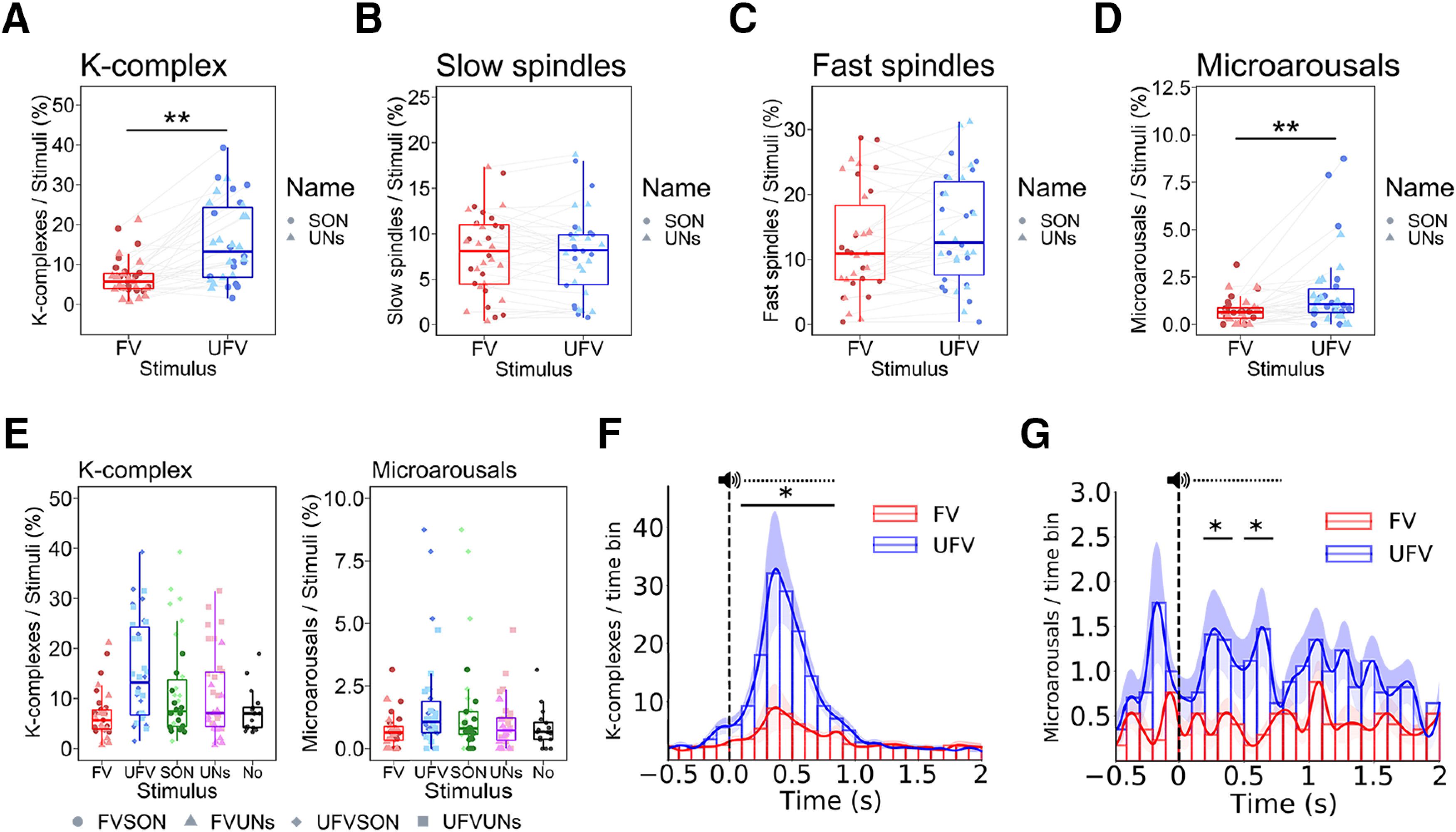
Selective sleep-specific responses to unfamiliar voices during NREM sleep. ***A***, Differences in the triggered K-complexes between the familiar voice (FV) and the unfamiliar voice (UFV) in the 2000 ms poststimulus-onset window. UFVs triggered more K-complexes than FVs. ***B***, ***C***, However, the numbers of (***B***) slow and (***C***) fast spindles did not differ between FV and UFVs. ***D***, Differences in triggered microarousals between FVs and UFVs demonstrating the higher number of microarousals triggered by UFVs. ***E***, The amount of triggered K-complexes and microarousals by all our stimulus categories and compared with those detected in the same number of 2000 ms no-stimulation epochs. ***F***, ***G***, Temporal aspects of the difference in the triggered K-complexes and microarousals. The difference between UFV and FV in the number of triggered K-complexes was significant from 100 ms to 800 ms (indicated by bar and asterisks; ***F***). The difference in the number of microarousals between FVs and UFVs was significant in the periods from 200 to 400 ms, and from 500 to 700 ms (***G***). Box plots show the median, and the whiskers depict the 25% and the 75% quartiles. Each dot/triangle represents one participant in one condition. Top, The dashed horizontal line represents the mean duration of our stimuli (808 ms), the lines depicts the means of the bins, and the shadings indicate the standard error of the mean (***F***, ***G***). *p < 0.05, **p < 0.001. FVSON, Familiar voice speaking the subject's own name; UFVSON, unfamiliar voice speaking the subject's own name; FVUNs, familiar voice speaking two unfamiliar names, UFVUNs, unfamiliar voice speaking two unfamiliar names.

We subsequently sought to investigate whether brain responses to auditory stimuli differ depending on their content, that is, the name and/or voice used in the stimulus. We used a nonparametric test from the nparLD package with two within factors, that is, *Name* (SON and UNs) and *Voice* (FV and UFV). KC responses to auditory stimuli showed a significant effect of *Voice*, as UFVs triggered more KCs than FVs (Fig. 6*A*; *ATS(1)* = 16.10, *p* > 0.001, *RTE_UFV_* = *0.76, RTE_FV_* = 0.24), no effect of *Name* (*ATS(1)* = 0.09, *p* = 0.76, *RTE_SON_* = 0.48, *RTE_UNs_* = 0.52), and a significant interaction *Name* × *Voice* (*ATS (1)* = 11.86, *p* = 0.001). *Post hoc* tests revealed that the amount of KCs triggered by the combination FV-SON was marginally higher than that triggered by the combination FV-UNs (*ATS(1)* = 4.17, *p_bonf_* = 0.08, *RTE_FVSON_* = 0.56, *RTE_FVUNs_* = 0.44), whereas there was no difference between UFV-SON and UFV-UNs (*ATS(1)* = 4.17, *p_bonf_* = 0.19, *RTE_UFVSON_* = 0.53, *RTE_UFVUNs_* = 0.47).

For fast and slow spindles, however, there was no effect of *Voice* (Fig. 6*B*,*C*; slow: *ATS(1,16)* < 0.001, *p* = 0.99, *RTE_UFV_* = 0.5, *RTE_FV_* = 0.5 − fast: *ATS(1,16)* = 2.71, *p* = 0.10, *RTE_UFV_* = 0.53, *RTE_FV_* = 0.47), no effect of *Name* (slow: *ATS(1,16)* = 0.46, *p* = 0.5, *RTE_SON_* = 0.51, *RTE_UNs_* = 0.49 − fast: *ATS(1,16)* = 0.62, *p* = 0.43, *RTE_SON_* = 0.49, *RTE_UNs_* = 0.51), and no interaction *Name* × *Voice* (slow: *ATS(1,16)* = 0.11, *p* = 0.74, *RTE_UFVSON_* = 0.51, *RTE_UFVUNs_* = 0.49, *RTE_FVSON_* = 0.52, *RTE_FVUNs_* = 0.48 − fast: *ATS(1,16)* = 2.63, *p* = 0.10, *RTE_UFVSON_* = 0.50, *RTE_UFVUNs_* = 0.55, *RTE_FVSON_* = 0.47, *RTE_FVUNs_* = 0.47). Microarousals showed a main effect of *Voice* (Fig. 6*D*; *ATS(1)* = 9.14, *p* = 0.002, *RTE_UFV_* = *0.59*, *RTE_FV_* = 0.41), no effect of *Name* (*ATS(1)* = 1.16, *p* = 0.29, *RTE_SON_* = 0.53, *RTE_UNs_* = 0.47) and no interaction *Name* × *Voice* (*ATS(1)* = 0.19, *p* = 0.66, *RTE_UFVSON_* = 0.61, *RTE_UFVUNs_* = 0.58, *RTE_FVSON_* = 0.44, *RTE_FVUNs_* = 0.37).

Figure 6*E* depicts the number of KCs and microarousals evoked by different stimulus types as compared with a 2000 ms no-stimulation intervals. Further, a temporally resolved analysis (see above, Statistical analyses) showed that the difference in the evoked KCs between FVs and UFVs occurred in the 100–800 ms poststimulus window (Fig. 6*F*; ∑*t*_(16)_ = 25.35, *p* = 0.002, *d* = 1.02). For microarousals (Fig. 6*G*), the difference appeared 200 ms poststimulus onset, that is, 100 ms later than that of KCs, and was significant between 200 and 700 ms poststimulus (200–400 ms: *t*_(16)_ = 5.92, *p* = 0.01, *d* = 0.84; 500–700 ms: *t*_(16)_ = 5.19, *p* = 0.02, *d* = 0.76). It is worth mentioning that we observed a transient increase in the number of microarousals ∼200 ms before the onset of UFV stimuli; however, this increase did not yield a significant statistical difference.

**Figure 7.**
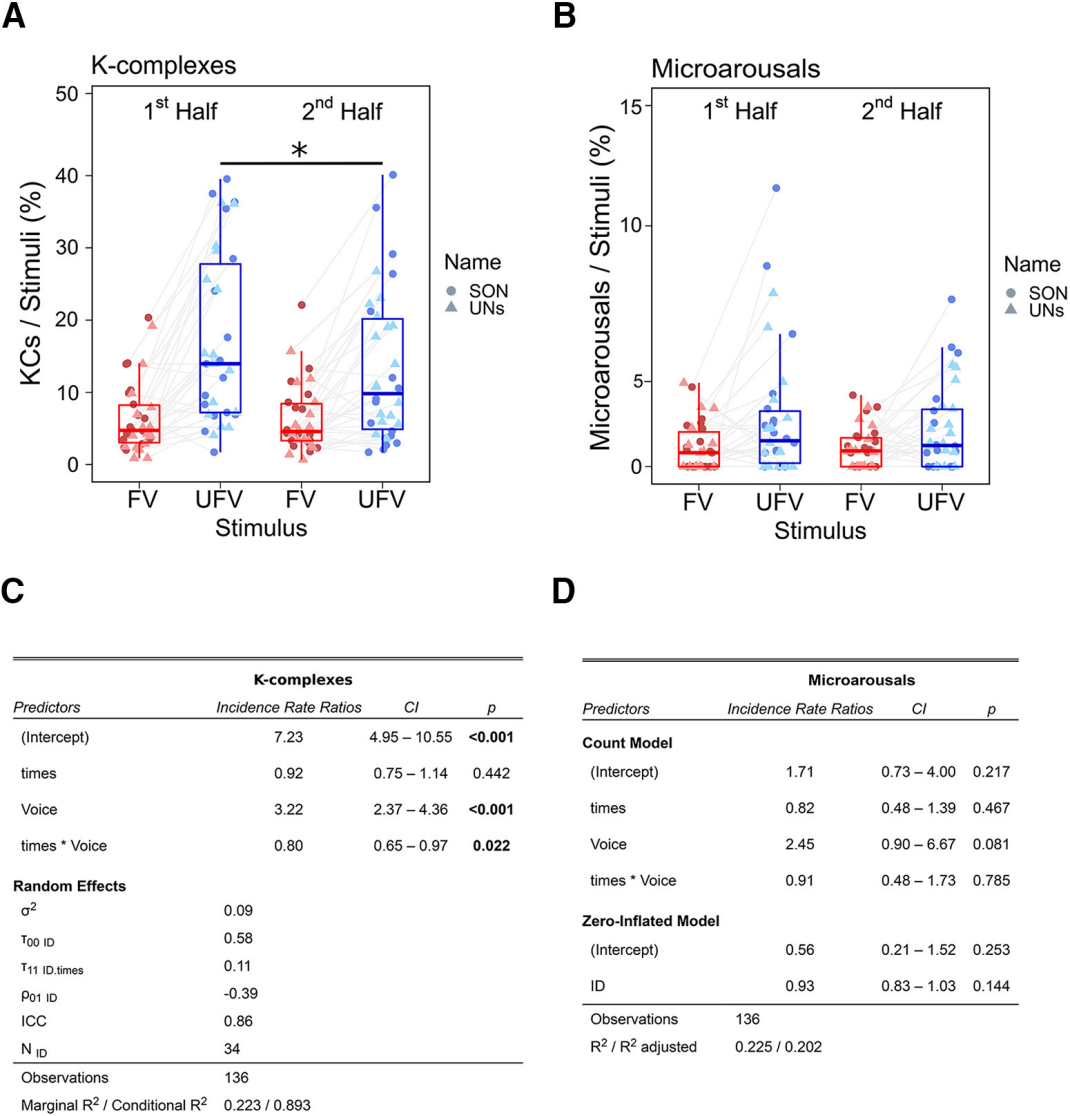
The effect of time on sleep-specific responses to unfamiliar voices during NREM sleep. ***A***, The difference in the numbers of triggered K-complexes to the familiar voice (FV) and unfamiliar voice (UFV) from the first to the second half of the night. A Generalized Linear Mixed Model (GLMM) using Poisson distribution revealed a significant interaction, *Time* × *Voice*, as only the amount of UFV-triggered K-complexes decreased in the second half. ***B***, A GLMM using zero-inflated Poisson distribution showed that the number of triggered microarousals did not change from the first to the second half of the night. ***C***, A statistical report of the GLMM model of K-complexes. We added Time (first half and second half), and Voices (familiar and unfamiliar voices) as fixed effects. Moreover, we assigned random intercepts and slopes for each subject. We found a significant main effect of voice, no effect of time, and a significant interaction, *Time* × *Voice*. ***D***, Statistical report of the zero-inflated Poisson GLMM of microarousals. Similar to K-complexes, we added Time (first half and second half), and Voices (familiar and unfamiliar voices) as fixed effects and random intercepts and slopes for each subject. No main effect of time and no interaction, *Time* × *Voice*, indicating that the amount of triggered microarousals did not change from the first to the second half of the night. *p < 0.05. Box plots show the median, and the whiskers depict the 25% and the 75% quartiles. Each dot/triangle represents one participant in one condition.

To illustrate whether the unfamiliarity of the voice is candidly the main cause of the difference in KC and microarousal responses, we divided the night into halves and hypothesized that brain responses to UFVs, but not to FVs, will decrease from the first to the second half, as the UFVs become more familiar with time. We modeled the change in the number of KCs from the first to the second half of the night using a GLMM with Poisson distribution fit by maximum likelihood (Fig. 7*A*). We found a main effect of *Voice* (*B̂* = 1.17 ± 0.16, *z* = 7.51, *p* < 0.001), no effect of *Time* (*B̂* = −0.08 ± 0.11, *z* = −0.77, *p* = 0.44), and a significant interaction *Time* × *Voice* (*z* = −2.28, *p* = 0.02). *Post hoc* tests revealed a significant decrease of the UFV-triggered KCs from the first to the second half of the night (*B̂* = −0.31 ± 0.08, *z* = 3.54, *p* = 0.002, *d* = 0.31), whereas FV-evoked KCs did not change (*B̂* = −0.08 ± 0.11, *z* = 0.77, *p* = 0.87, *d* = 0.08). Conversely, for microarousals, a GLMM with zero-inflated Poisson distribution (Fig. 7*B*) demonstrated a marginally significant effect of *Voice* (*B̂* = 0.89 ± 0.51, *z* = 1.75, *p* = 0.08), no effect of *Time* (*B̂* = −0.19 ± 0.27, *z* = 0.73, *p* = 0.98), and no interaction *Time* × *Voice* (*B̂* = −0.09 ± 0.33, *z* = −0.27, *p* = 0.55). We provide a more detailed description of the GLMM results in Figure 7*C*,*D*.

### UFVs evoke stronger K-complex-mediated brain responses during NREM sleep

**Figure 8.**
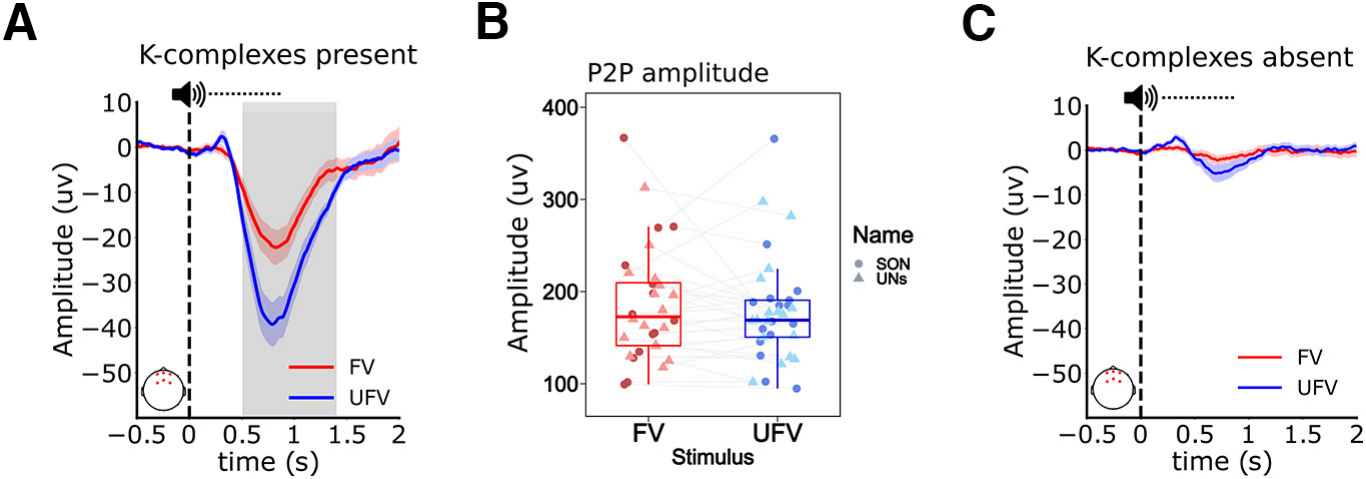
Unfamiliar voices elicited stronger brain responses in the presence of the evoked K-complex. ***A***, ERP contrast between familiar voice (FV) and unfamiliar voice (UFV) in the presence of the auditory-evoked K-complexes. UFVs triggered a larger amplitude of the evoked response between 510 ms and 1400 ms as shown by the gray shadings. Extended Data Figures 8-1 and 8-2 illustrate the methodological underpinnings of the discrepancies between the amplitude and latency of the negative component in ***A*** as compared with the conventional amplitude and latency of the N550 in the literature. ***B***, Comparison of the peak-to-peak amplitude (P2P; N550 to P900) of the evoked K-complexes showing no difference in the amplitudes of the evoked K-complexes between FVs and UFVs. ***C***, ERP responses to FVs and UFVs in the absence of evoked K-complexes. There was no difference in the ERP amplitudes between FVs and UFVs when no K-complexes were evoked. The solid blue and red lines and the shadings represent the mean and the standard error of the mean, respectively. Top, The dashed horizontal line represent the mean duration of our stimuli (808 ms). Vertical dashed lines (at *x* = 0) represent stimulus onset. Bottom left, The red dots on the topographical plots indicate the locations of the channels used for the analysis. Box plots show the median, and the whiskers depict the 25% and the 75% quartiles. Each dot/triangle represents one participant in one condition.

Next, we aimed to examine the neural dynamics underlying the aforementioned differences in sleep microstructure. Therefore, we compared the ERPs between FVs and UFVs in the following conditions: (1) when the stimuli triggered KCs and (2) when no KCs were triggered. We found that when KCs were triggered, UFVs evoked a larger, more pronounced negative peak (Fig. 8*A*; ∑*t*_(16)_ = −436.35, *p* > 0.001, d = 0.96) that resembles the N550 of the KC in its temporal and morphologic characteristics. Importantly, however, we observed that the peak of the N550 potential occurred later (∼750–800 ms) than the usual time window (500–550 ms) and had a much smaller amplitude (20–50 µV) than that (∼100 µV) previously reported in the literature (Colrain, 2005; Halász, 2005; Laurino et al., 2014). We illustrate that these discrepancies with the previous literature are because of the relatively large temporal window we defined for the detection of evoked KCs (2000 ms) as well as our decision to use an average reference compared with the mastoid reference used in earlier studies (Bastien and Campbell, 1992, 1994; Colrain, 2005; Halász, 2005; Laurino, 2014). Extended Data Figures 8-1 and 8-2 show the difference in the amplitude of the N550 according to the different referencing procedures as well as the latency jitter of the N550 peak on the single-trial level. Further, to confirm that the difference in the ERPs does not reflect a difference in the amplitude of the evoked KCs, we compared the peak-to-peak amplitudes of the evoked KCs, as measured by the detection algorithm, between FVs and UFVs (Fig. 8*B*). Peak-to-peak amplitudes showed no significant effect of *Voice* (*ATS(1)* = 1.47, *p* = 0.23, *RTE_UFV_* = 0.48, *RTE_FV_* = 0.52), *Name* (*ATS(1)* = 0.71, *p* = 0.39, *RTE_SON_* = 0.52, *RTE_UNs_* = 0.48), and no interaction *Voice* × *Name* (*ATS(1)* = 1.47, *p* = 0.23, *RTE_UFVSON_* = 0.49, *RTE_UFVUNs_* = 0.47, *RTE_FVSON_* = 0.54, *RTE_FVUNs_* = 0.50). Intriguingly, when no KCs are evoked, we found no difference in the amplitude of the ERPs between UFVs and FVs (Fig. 8*C*; 0.3−0.37 s: ∑*t*_(16)_ = 27.31, *p* = 0.13).

10.1523/JNEUROSCI.2524-20.2021.f8-1 Figure 8-1 K-complex trials referenced to contralateral mastoids. Top row, The comparison between the evoked K-complexes to FVs and UFVs centered on the start of the K-complex, that is, negative-going zero-crossing of the K-complex. In more details, we extracted the K-complexes that were detected by our algorithm in the 2000 ms poststimulus window and created epochs from −0.5 s to 2s around the start of the K-complex events (negative going zero crossing). Bottom row, The trials where a K-complex was evoked, centered on the start of the stimulus (0 = stimulus onset). We show the grand average ERP over all subjects (left). Note that the amplitude of the negative wave exceeds 100 UV and that there is no difference in the amplitude of the ERPs between FVs and UFVs. We also show the single-subject data of the K-complex averaged over trials for each subject (middle). Finally, we show single trials (*n* = 20 trials per condition) from one subject (right). Together, this figure corroborates our claim that the difference in the ERPs between FVs and UFVs shown in Figure 8*A* is due to difference in time locking rather than the amplitude of the evoked K-complexes. K-complexes were detected at C3. We show data from one electrode F3, referenced to the contralateral mastoid (A2). Shadings reflect the SEM. Vertical dashed lines (at *x* = 0) represent the start of the evoked K-complex detected by our algorithm (top) or stimulus onset (bottom). Shadings represent SEM. Download Figure 8-1, EPS file.

10.1523/JNEUROSCI.2524-20.2021.f8-2 Figure 8-2 K-complex trials referenced to an average reference. Similar to Extended Data Figure 8-1, in the top row we show the evoked K-complexes to FV and UFV centered on start of the K-complexes (the zero crossing of the signal from positive to negative), whereas the bottom row depicts the trials when a K-complex was evoked centered around the start of the stimuli. The difference between Extended Data Figures 8-1 and 8-2 is the choice of the reference. Note the decrease in the amplitude of the ERPs from Extended Data Figures 8-1 to 8-2 due to changing the reference (from a mastoid reference in Extended Data Figure 8-1 to an average reference in Extended Data Figure 8-2). Left, Grand average ERPs over all subjects for both conditions FVs and UFVs. Middle, Single-subject data for the K-complex trials averaged over all trials. Right, Single trial data (*n* = 20 per condition) from one subject. K-complexes were detected at C3. We show ERPs from one electrode (F3) referenced to an average reference. Vertical dashed lines (at *x* = 0) represent the start of the K-complex event (top) or stimulus onset (bottom). Shadings represent SEM. Download Figure 8-2, EPS file.

### K-complex-mediated brain responses to UFVs reflect sensory processing

**Figure 9.**
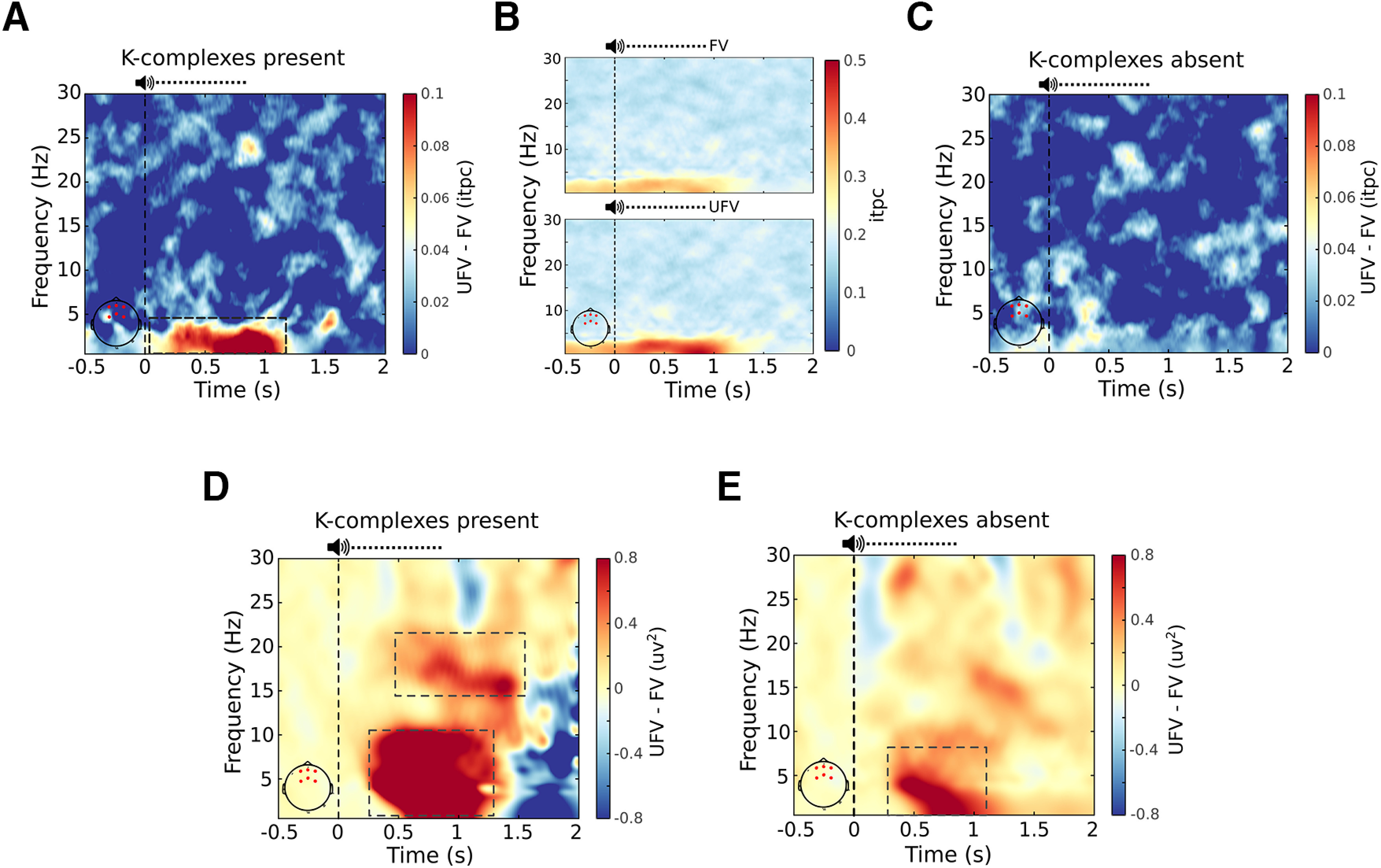
Brain responses to unfamiliar voices in the presence of the evoked K-complex reflect further processing. ***A***, The difference in intertrial phase coherence (ITPC) values between the familiar voice (FV) and the unfamiliar voice (UFV) in the presence of the evoked K-complex. UFVs evoked significantly higher ITPC than FV in the delta (1–4 Hz) frequency band. Note that the largest difference in phase locking overlaps with the difference between the ERPs in Figure 8*A*. ***B***, Separate ITPC plots for FVs (top) and UFVs (bottom) showing stronger ITPC values following UFVs and indicating that the difference in ITPC values is because of an increase in ITPC following UFVs. ***C***, ITPC difference between UFVs and FVs in the absence of the evoked K-complexes. There is no difference in ITPC between FVs and UFVs in the absence of evoked K-complexes. ***D***, ***E***, Spectral power maps of the power differences between FVs and UFVs. We demonstrate stronger responses to UFV in a broad frequency range (∼1–10 Hz) regardless of the presence (***D***) or the absence (***E***) of K-complexes. However, stronger high-frequency (>16 Hz) responses to UFVs appeared only in the presence of K-complexes. Top, The dashed horizontal line represents the mean duration of our stimuli (808 ms). Vertical dashed lines (at *x* = 0) represent stimulus onset. Bottom left, The red dots on the topographical plots indicate the locations of the channels used for the analysis.

We then speculated that the difference in the amplitude of the ERPs, if not because of a difference in the amplitude of the evoked KCs, might reflect more synchronized evoked responses to UFVs as compared with FVs. Therefore, we compared the phase consistency of brain responses between FVs and UFVs via the ITPC metric (Tallon-Baudry et al., 1996). We observed stronger ITPC following UFVs as compared with FVs in the delta band (1–4 Hz; Fig. 9*A*; ∑*t*_(16)_ = 2413.98, *p* = 0.003, *d* = 1.06) indicating more synchronized brain responses to UFVs. Figure 9*B* shows the ITPC contrast between FVs and UFVs indicating that difference in the IPTC is a product of stronger time-locked responses to UFVs. Similar to our ERP analysis, in the absence of the evoked KCs, IPTC values did not differ between FVs and UFVs (Fig. 9*C*; ∑*t*_(16)_ = 2521.7, *p* = 0.001).

Finally, we performed spectral analysis of brain responses to UFVs and FVs in the presence and absence of evoked KCs. We observed stronger low-frequency response (delta band; 1–4 Hz) to UFVs as compared with FVs (Fig. 9*D*,*E*) that started ∼250 ms poststimulus onset. This delta response appeared independent of the presence (Fig. 9*D*; ∑*t*_(16)_ = 9395.64, *p* < 0.001, *d* = 1.25) or absence (Fig. 9*E*; ∑*t*_(16)_ = 4097.56, *p* = 0.013, *d* = 0.86) of the evoked KC.

Crucially, only in the presence of KCs, UFVs additionally elicited a significant increase in the power of higher frequencies (>16 Hz) as compared with FVs (Fig. 9D, ∑*t*_(16)_ = 5020.62, *p* = 0.006, *d* = 1.04) starting ∼500 ms poststimulus.

## Discussion

In this study, we presented sleepers with their own first names (SON) and two UNs spoken by either an FV or a UFV during a full night of sleep with polysomnography. We show that although auditory stimulation did change sleep architecture (Fig. 4*A*–*C*), it induced prominent, stimulus-specific changes in sleep microstructure. Generally, presenting auditory stimuli during NREM sleep increased the number of KCs, spindles, and microarousals (Fig. 5). UFVs triggered more KCs (Fig. 6*A*) and microarousals (Fig. 6*D*) than FVs. However, we found no difference in the amount of triggered KCs, spindles, or microarousals between SONs and UNs. The difference in the numbers of evoked KCs and microarousals between FVs and UFVs appeared ∼100 ms poststimulus (200 ms for microarousals) and extended over the whole duration of the stimuli ∼800 ms (Fig. 6*F*,*G*). Although the number of the UFV-triggered KCs decreased in the second half of the night, the numbers of evoked microarousals remained relatively stable throughout the night (Fig. 7). Moreover, in the presence of the auditory-evoked KC, UFVs triggered larger evoked responses (Fig. 8*A*), which did not reflect a difference in the amplitudes of the evoked KCs (Fig. 8*B*,*C*) but rather more synchronized brain responses to UFVs relative to FVs (Fig. 9*A*,*B*). Similarly, brain responses to UFVs demonstrated an increase in the power of high frequencies (>16 Hz), suggesting a stronger arousal reaction to UFVs (Fig. 9*D*). Crucially, we were not able to detect such differential brain responses between FVs and UFVs in the absence of the evoked KC.

It has previously been suggested that the more relevant the stimulus, the higher its tendency to trigger KCs (Halász, 2005). In this regard, our results pose UFVs as more relevant—or in evolutionary terms potentially more threatening (Blume et al., 2018)—and consequently more arousing to the sleeper than FVs. Indeed, the increase in microarousals following UFVs suggests a transient shift toward external processing of vital environmental stimuli. In the same vein, the decrease in the number of UFV-evoked KCs in the second half compared with the first half of the night (Fig. 7*A*) supports the notion that the sleeping brain continues to learn new information during sleep (Züst et al., 2019). That is, homeostatic regulatory processes alone cannot explain this observation because the number of FV-evoked KCs did not change, indicating a stimulus-specific attenuation of brain responses. It might be that the sleeping brain learns, through repeated processing, that an initially unexpected stimulus poses no immediate threat to the sleeper and consequently decreases its response to it. Conversely, in a safe sleep environment, the brain might be expecting to hear FVs and consistently inhibits any response to such stimuli to preserve sleep (Fig. 6*E*). Although this assumption remains speculative, it entails a thorough investigation of the ability of the brain to generate top-down predictions of the the external sensory world during sleep. Nevertheless, our results suggest that the unfamiliarity of voice is a strong promoter of brain responses during NREM sleep.

What is the role of the auditory-evoked brain responses during NREM sleep? Central to such responses is the KC, that is, the most prominent sleep-specific response to sensory stimulation. To answer this question, we contrasted trials during which FVs and UFVs triggered KCs. In such trials, UFVs evoked a larger negative potential that resembles the N550 component of the KC (Fig. 7*A*). The N550 has been associated with large-scale neuronal silencing that protects sleep (Cash et al., 2009; Laurino et al., 2014) and, conversely, an arousal reaction that facilitates stimulus processing (Atienza et al., 2001). In our study, the amplitudes of the evoked KCs did not differ between FVs and UFVs, corroborating the previous literature presenting the evoked KC as an all-or-none phenomenon (Bastien and Campbell, 1992). Rather, the difference in the N550 amplitude between FVs and UFVs was because of more synchronized brain responses to UFVs as indicated by the ITPC values (Fig. 9*A*). Stimulus-induced phase modulations have been suggested to promote information processing and transmission in the cortex (Lakatos et al., 2013; Canavier, 2015; Voloh and Womelsdorf, 2016), and increased ITPC values have been associated with better cognitive performance (Hanslmayr et al., 2005; Eidelman-Rothman et al., 2019) and enhanced attention (Joon Kim et al., 2007) during wakefulness. Together, our results suggest that the preferential brain responses to UFVs during NREM sleep reflect sensory processing. Importantly, the shorter poststimulus time window during which UFVs elicited KCs as compared with FVs, indicated by the narrower peak in Figure 6*F*, implies better temporal alignment of KCs following UFVs and emphasizes the contribution of the evoked KCs to the observed phase modulations. Finally, the stronger high-frequency responses (>16 Hz; Fig. 9) to UFVs that appeared only in the presence of KCs corroborate the role of the auditory-evoked KCs in promoting sensory processing of relevant information. Together, our findings suggest a central role for KCs in the extraction and processing of relevant external sensory information during NREM sleep.

We did not find any differences in brain responses between SON and UNs in contrast to the previous literature (Oswald et al., 1960; Perrin et al., 1999). One explanation might be that although sleep preserves low-level auditory processing, it attenuates higher order linguistic tracking (Makov et al., 2017). We speculate that this is because of the disruption of the activity of the large-scale networks necessary for higher order name processing (Démonet et al., 1992; Luke et al., 2002) because of the loss of long-range cortical connectivity during sleep (Massimini et al., 2005). Further research should elucidate the mechanisms of language tracking during sleep.

We show that the auditory-evoked spindles are not influenced by specific characteristics of the auditory stimuli (i.e., name or voice). However, the role of spindles in response to sensory stimuli during sleep is far from clear. Previous research has shown that spindles attenuate or even inhibit the processing of auditory information (De Gennaro and Ferrara, 2003; Schabus et al., 2012; Blume et al., 2018). Other work suggests that brain responses are preserved during spindles (Sela et al., 2016) and even argues for a role for spindles in the processing of memory-related sensory information (Antony et al., 2018; Cairney et al., 2018; Jegou et al., 2019). More research should investigate the role of spindles in response to sensory information irrelevant to ongoing memory processes.

Finally, we found no relevant changes in sleep macrostructure and architecture because of auditory stimulation (Fig. 4). In fact, microarousals represent an integral part of healthy sleep as they ensure its reversibility (Halász et al., 2004). Hence, their slight increase should not be viewed as a disruption to normal sleep. Further, the analysis of microstates even indicates a shift toward deeper sleep in response to UVF stimuli (Fig. 4*C*), which is most likely a by-product of more auditory-evoked KCs that influenced the staging. However, the classical 30 s sleep staging might not be sensitive enough to capture subtle changes in sleep microstructure. Hence, the development and refinement of new fine-grained methods, such as the Hori-based microstate classification (Jagannathan et al., 2018) promises better monitoring of transient sleep fluctuations, especially in the presence of sensory perturbation.

In summary, sleep appears to be far from a homogenous state of unconsciousness. There are temporal windows in sleep during which the brain filters, extracts, and processes relevant external information. We speculate that such content-specific, dynamic reactivity to sensory signals enables the brain to enter a sentinel processing mode (Blume et al., 2018) during which it preserves the ability to efficiently engage in the important internal processes that are ongoing during sleep while remaining connected to the surrounding environment.
